# Paternal and maternal bonding styles in childhood are associated with the prevalence of chronic pain in a general adult population: the Hisayama Study

**DOI:** 10.1186/s12888-015-0574-y

**Published:** 2015-07-31

**Authors:** Kozo Anno, Mao Shibata, Toshiharu Ninomiya, Rie Iwaki, Hiroshi Kawata, Ryoko Sawamoto, Chiharu Kubo, Yutaka Kiyohara, Nobuyuki Sudo, Masako Hosoi

**Affiliations:** 1 Department of Psychosomatic Medicine, Kyushu University Hospital, 3-1-1 Maidashi, Higashi-ku, Fukuoka, 812-8582 Japan; 2 Department of Environmental Medicine, Graduate School of Medical Sciences, Kyushu University, Fukuoka, 812-8582 Japan; 3 Division of Research Management, Center for Cohort Studies, Graduate School of Medical Sciences, Kyushu University, Fukuoka, 812-8582 Japan; 4 Department of Psychosomatic Medicine, Graduate School of Medical Sciences, Kyushu University, Fukuoka, 812-8582 Japan

**Keywords:** Affectionless control, Care, Chronic pain, Optimal bonding, Overprotection, Parental bonding, Population, Prevalence

## Abstract

**Background:**

Previous research has suggested that extraordinary adverse experiences during childhood, such as abuse, are possible risk factors for the development of chronic pain. However, the relationship between the perceived parental bonding style during childhood and chronic pain has been much less studied.

**Methods:**

In this cross-sectional study, 760 community-dwelling Japanese adults were asked if they had pain that had been present for six months or more. They completed the Parental Bonding Instrument (PBI), a self-administrated questionnaire designed to assess perceived parental bonding, and the Patient Health Questionnaire-9 to assess current depressive symptoms. The PBI consists of care and overprotection subscales that are analyzed by assigning the parental bonding style to one of four quadrants: Optimal bonding (high care/low overprotection), neglectful parenting (low care/low overprotection), affectionate constraint (high care/high overprotection), and affectionless control (low care/high overprotection). Logistic regression analysis was done to estimate the contribution of the parental bonding style to the risk of chronic pain, controlling for demographic variables.

**Results:**

Compared to the optimal bonding group, the odds ratios (ORs) for having chronic pain were significantly higher in the affectionless control group for paternal bonding (OR: 2.21, 95 % CI: 1.50-3.27) and for maternal bonding (OR: 1.60, 95 % CI: 1.09-2.36). After adjusting for depression, significance remained only for paternal bonding.

**Conclusion:**

The results demonstrate that the parental bonding style during childhood is associated with the prevalence of chronic pain in adults in the general population and that the association is more robust for paternal bonding than for maternal bonding.

## Background

Chronic pain is a major health care problem that has a considerable impact on human suffering and enormous economic implications for society. Although the definition of chronic pain is not uniform across epidemiological studies of pain, its reported prevalence in the general population ranges from 11 to 55 % [1, 2]. Chronic pain is known to be a complex biopsychosocial condition influenced by a wide range of psychosocial factors, such as beliefs about pain, pain-related fear, self-efficacy, psychological distress (catastrophizing, anxiety, depression), work-related problems, compensation status, and lack of social support [2, 3]. Therefore, in order to minimize the negative impact of chronic pain on the quality of life, it is important to identify the psychosocial factors associated with the development and persistence of chronic pain.

Adverse experiences during childhood are possible risk factors for the development and persistence of chronic pain. In fact, several studies have reported a positive relationship between childhood physical or sexual abuse and several chronic pain-related problems in adulthood [4–6]. In addition, a number of studies have also demonstrated that adverse experiences in childhood, other than abuse, are predictive of the development of chronic pain. For example, one longitudinal study reported that adverse childhood physical and psychological experiences, such as hospitalization following a traffic accident, institutional care, and the death of the mother, increased the risk of developing chronic widespread pain as an adult [7]. Moreover, these associations were not explained by concurrent psychological distress or social class in adulthood. Thus, it seems likely that extraordinary adverse events during childhood are related to the development of chronic pain later in life; however, the relation to childhood-related factors in daily life are largely unknown.

Bowlby [8] proposed that the early relationship between the parent and child (i.e., “attachment”) plays a crucial role in normal childhood development and long-term functioning. A child who has a history of secure attachment to parents usually grows to be a secure, self-reliant, and cooperative adult. On the other hand, if parents fail to meet the child’s needs, normal childhood development is adversely affected, leading to the expression of the maladaptive personality characteristics and mental disorders such as depression that are often found in patients with chronic pain. A growing body of empirical evidence suggests that adult attachment styles are related to pain-related variables. In pain free individuals, insecure attachment is associated with lower pain threshold and higher pain related distress [9, 10]. In samples of patients with a chronic pain condition, those with insecure attachment reported significantly higher levels of pain intensity, disability, and pain related suffering and lower level of pain self-efficacy [11–13]. These studies suggest that attachment style is related to adjustment to a pain experience and a vulnerability to the development of chronic pain conditions.

Parker reviewed attachment studies in order to identify key parental behaviors and attitudes that affect the formation of the attachment styles of their children [14]. Accordingly, they found that parental “care” and “overprotection” were two main factors that can affect bonding experiences with parents. It is reasonable to speculate that parental care and overprotection during childhood could affect the development and exacerbation of chronic pain later in life. However, there are few studies that address this issue in a community sample.

In the present study, we used the Parental Bonding Instrument (PBI), a self-report questionnaire developed to evaluate parental behavior styles, to examine if parental care and overprotection during childhood is related to the prevalence of chronic pain in a general adult population [14].

## Methods

### Participants

The Hisayama study, an epidemiological study of cerebrovascular and cardiovascular diseases, was established in 1961 in Hisayama Town, a suburb of the Fukuoka metropolitan area on the island of Kyushu in Japan. Surveys of the entire community regarding the health status of residents aged ≥40 years have been repeated every five years since 1961 [15]. In 2011, a cross-sectional survey was done for the present study. Data for the current analyses were obtained from survey responses to questions regarding pain and psychosocial variables. Of 2250 residents who responded, 840 (37.3 %) consented to participate in this study. After excluding the respondents (n = 56) who did not fully complete the questionnaire or who rated only one or none of their parents on the PBI due to parental death or no contact with either parent in childhood (n = 24), the data of 760 (286 men and 474 women) were available for analysis. This study was approved by the Kyushu University Institutional Review Board for Clinical Research, and written informed consent was obtained from all of the participants.

### Measures

#### Demographic variables

Age, sex, marital status, and education level were collected as background information. Marital status was classified as never married, divorced, separated, widowed, married, or cohabiting. The educational level was classified as follows: ≤9 years, 10–12 years, and >12 years.

#### Assessment of parental bonding

**Fig. 1:**
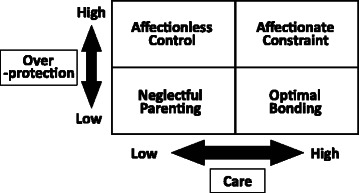
Parental bonding styles by PBI quadrant

Perceived parental bonding was measured using the Parental Bonding Instrument (PBI), which is based on the participants’ memories of their parents during the first 16 years after birth [14]. The PBI is a self-report questionnaire that consists of 25 items, including two principal dimensions, “care” and “overprotection”. The “care” dimension reflects perceived parental warmth, affection, and involvement in contrast to coldness, indifference, and rejection. Examples of the items include ‘Appeared to understand my problems and worries’ and ‘Frequently smiled at me’. The “overprotection” dimension reflects perceived parental psychological control and intrusion in contrast to the encouragement of autonomy and independence. Examples of the items include ‘Felt I could not look after myself unless she/he was around’ and ‘Tried to control everything I did’. The participants were asked to score their father’s and mother’s attitudes separately using a 4-point scale. Parental bonding evaluated by the PBI can be classified into four quadrants according to the possible combinations of the two dimensions: “Optimal bonding” (high care, low overprotection), “neglectful parenting” (low care, low overprotection), “affectionate constraint” (high care, high overprotection), and “affectionless control” (low care, high overprotection) (Fig. 1).

The PBI score reflects the actual parenting attitude, based on studies that used corroborative witnesses and independent observers [16, 17]. The PBI has long-term stability [18], and its subscales have a high level of test-retest reliability and internal consistency [19]. The Japanese version of the PBI has also been shown to have adequate validity [20]. For the present study, the respective internal consistencies (Cronbach’s coefficient alpha) of the care and overprotection subscales were 0.91 and 0.83 for paternal bonding and 0.88 and 0.83 for maternal bonding.

#### Assessment of depression

Depression was measured using the Patient Health Questionnaire-9 (PHQ-9), a self-report questionnaire that has been shown to be a reliable and valid assessment tool for both the diagnosis of depression and the evaluation of depression severity in primary care settings [21]. The PHQ-9 assesses the symptoms of depression over the past two weeks using nine questions rated on a 4-point scale; 0 (not at all) to 3 (nearly every day). These questions are based on the Diagnostic and Statistical Manual of Mental Disorders, fourth edition (DSM-IV) diagnostic criteria for major depression. Examples of items include ‘Little interest or pleasure in doing things’ and ‘Feeling down, depressed, or hopeless’. The sum of the nine items is calculated to obtain a depression score ranging from 0 to 27. Depression scores are categorized into three groups; minimal (<5), mild (5–9), and moderate to severe (>9). The PHQ-9 is useful for the evaluation of the depressive state of persons with common diseases and chronic pain-related conditions [22, 23]. The validity of the Japanese version of the PHQ-9 has been confirmed [24].

#### Assessment of chronic pain

Participants were first asked if they were experiencing any pain and then were asked about the duration of the pain. Chronic pain was defined as pain that had been present continuously or intermittently for ≥6 months. Participants were also asked to rate the average intensity of their pain in the past week on a visual analog scale (VAS). The anchors were “No pain” (0 mm) and “Pain as bad as it could be” (100 mm). The VAS for pain has been shown to be a reliable and valid measure of pain intensity [25]. Participants with pain identified their primary pain site from a list of ten sites according to the International Association for the Study of Pain site categories [26]: Head and face, neck, shoulder and upper limbs, thoracic region, abdomen, lower back, lower limbs, pelvic region, perineal and genital region, and multiple sites.

### Statistical analysis

Each PBI subscale was divided into three levels based on the tertiles of the scores from the total study sample. The prevalence of chronic pain was tested for trends across each of the PBI subscale score levels using logistic regression analysis. In order to account for the interaction between the care and overprotection subscales, each subscale was dichotomized at its cut-point and combined. Thereafter, paternal and maternal bonding was classified into quadrants. The quadrants of parental bonding were used to compare the prevalence of chronic pain by each parent. Multivariable logistic regression analysis was then done to estimate the contribution of the parental bonding style to the risk of chronic pain, while controlling for the demographic variables. Additionally, we adjusted for the PHQ-9 score to estimate the influence of depression on the association between a parental bonding style and chronic pain. The “optimal bonding” quadrant of parental bonding style was used as the reference category when estimating the risk of chronic pain in the other three quadrants. These analyses were done for men and women separately. Tests for a statistical interaction between the parental bonding style and sex of the participants were conducted by entering interaction terms for the quadrants of parental bonding and the sex of the participants in multivariate model. For all data handling and statistical tests, SPSS v17.0 for Windows (SPSS Inc., Chicago, USA) was used.

## Results

Table 1 summarizes the characteristics of the study sample. The prevalence of chronic pain was 46.4 % (43.4 % for men, 48.3 % for women, *P* = 0.20). The primary pain sites were the lower back (26 %), lower limbs (26 %), upper limbs (25 %), neck (9 %), head (4 %), upper back (3 %), and other sites (8 %). The median pain intensity was 40 mm (interquartile range, 20-58 mm). The correlation coefficient between the paternal and maternal care scores was 0.69 (*P* < 0.001) and that between paternal and maternal overprotection was 0.83 (*P* < 0.001).

**Table 1:** Characteristics, PBI scores, and chronic pain prevalence

Variable	n = 760	
Sociodemographic characteristics		
Age (years)	59.3	±11.5
	(range 39-92)	
Women (%)	62.4	
Marital status (%)		
Never married	5.7	
Married/Cohabiting	80.9	
Divorced/Separated	3.8	
Widowed	9.6	
Education level (%)		
≤9-years	13.6	
10-12 years	51.8	
>12 years	34.6	
PHQ-9 score (%)		
Minimal (<5)	74.0	
Mild (5–9)	21.7	
Moderate to severe (≥10)	4.3	
PBI score		
Paternal care	28.0	(22-33)
Paternal overprotection	8.0	(4-13)
Maternal care	31.0	(26-35)
Maternal overprotection	8.0	(3-13)
Chronic pain (%)	46.4	

Values are means ± standard deviation, frequencies, or median (interquartile range)

*PHQ-9* Patient Health Questionnaire-9, *PBI* Parental Bonding Instrument

**Fig. 2:**
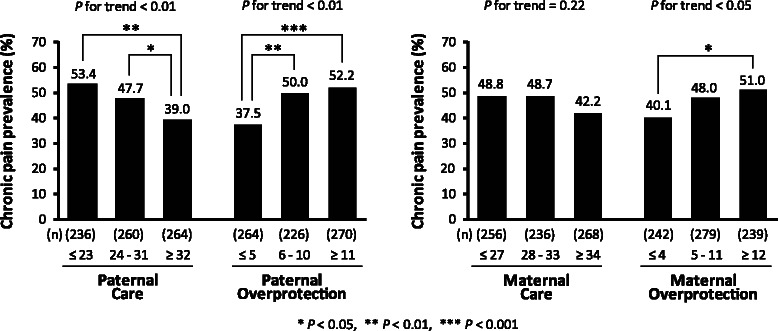
Chronic pain prevalence according to PBI score (tertiles of the subscales). *P* for trend: *P* value of the test for linear relationships between the parenting categories and chronic pain prevalence

Figure 2 shows the prevalence of chronic pain for each PBI subscale score. Paternal care was negatively associated with the prevalence of chronic pain, in contrast to paternal overprotection, which was positively associated. Maternal overprotection also showed a positive association with the prevalence of chronic pain, whereas no significant association was noted for maternal care (*P* for trend = 0.22).

In this study, the optimal parenting style for chronic pain is defined as a combination of the highest tertile of care and lowest tertile of overprotection by both parents, as shown in the data of Fig. 2 and as illustrated in Fig. 1, because the participants who belonged to both tertiles had the lowest prevalence of chronic pain. Binary variables were generated using these tertile cut-points. The cut-points for the paternal care and overprotection scores were 31 and 5 points, respectively. For maternal bonding, the care and overprotection cut-points were 33 and 4 points, respectively. Scores higher than the cut-points were defined as “high”, while scores equal to or below the cut-points were defined as “low” in the current study.

**Fig. 3:**
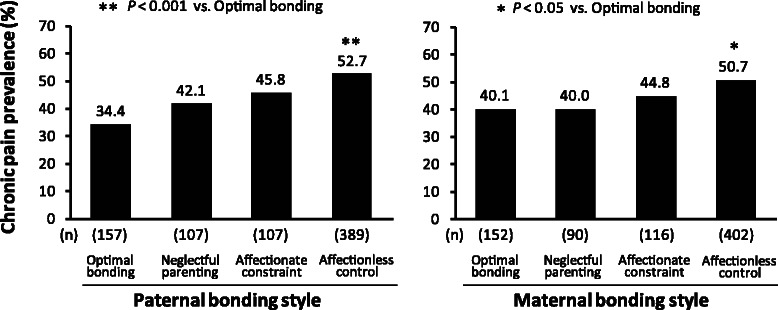
Chronic pain prevalence according to the PBI quadrant

Figure 3 illustrates the prevalence of chronic pain according to the PBI quadrant, as described in Fig. 1. The affectionless control group had the highest chronic pain prevalence of the four groups for both the paternal and maternal bonding styles. On the other hand, the prevalence of chronic pain in the optimal bonding group was the lowest of the four paternal bonding styles and was significantly lower than that of the affectionless control group. With regard to the maternal bonding styles, the prevalence of chronic pain in the affectionless control group was significantly higher than that in the optimal bonding group.

Table 2 shows the odds ratios (ORs) for the likelihood of chronic pain according to the paternal or maternal bonding style. The OR of the affectionless control group was significantly higher than that of the optimal bonding group for both the paternal and maternal bonding styles. These differences were not substantially affected after adjusting for demographic variables. A more distinct difference in the OR for chronic pain was observed for the paternal bonding styles (OR: 2.21, 95 % CI: 1.50-3.27) than for the maternal bonding styles (OR: 1.60, 95 % CI: 1.09-2.36). After adjustment for depression, the difference in the ORs for the paternal bonding styles was reduced but remained significant, whereas the difference in the ORs for the maternal bonding styles was no longer significant.

**Table 2:** Odds ratios for chronic pain according to the PBI quadrant

Parental bonding style	Number of subjects	Number with chronic pain	Crude	*P* value	Multivariable-adjusted (demographic)^a^	*P* value	Multivariable-adjusted (demographic and depression)^b^	*P* value
			OR (95 %CI)		OR (95 %CI)		OR (95 %CI)	
Father								
Optimal parenting	157	54	1.00 (reference)		1.00 (reference)		1.00 (reference)	
Neglectful parenting	107	45	1.38 (0.84-2.30)	0.207	1.47 (0.88-2.46)	0.138	1.41 (0.84-2.37)	0.192
Affectionate constraint	107	49	1.61 (0.97-2.67)	0.063	1.61 (0.97-2.67)	0.066	1.51 (0.91-2.53)	0.114
Affectionless control	389	205	2.13 (1.45-3.12)	<0.001	2.21 (1.50-3.27)	<0.001	1.80 (1.21-2.70)	0.004
Mother								
Optimal parenting	152	61	1.00 (reference)		1.00 (reference)		1.00 (reference)	
Neglectful parenting	90	36	1.00 (0.58-1.69)	0.984	1.01 (0.59-1.73)	0.964	1.00 (0.58-1.73)	0.999
Affectionate constraint	116	52	1.21 (0.74-1.98)	0.441	1.29 (0.79-2.12)	0.314	1.26 (0.76-2.08)	0.371
Affectionless control	402	204	1.54 (1.05-2.24)	0.026	1.60 (1.09-2.36)	0.016	1.31 (0.88-1.96)	0.181

*OR* odds ratio, *CI* confidence interval

^a^Multivariable adjustment was done for age, sex, marital status, and years of education

^b^Multivariable adjustment was done for age, sex, marital status, years of education, and depression

Additionally, the bonding styles were divided as optimal and non-optimal to determine the prevalence of chronic pain according to the combination of paternal (optimal or non-optimal) and maternal (optimal or non-optimal) bonding. In comparison with the combination paternal optimal/maternal optimal, significant differences were found for the paternal non-optimal/maternal optimal (OR: 2.66, 95 % CI: 1.28-5.54, *P* = 0.009) and paternal non-optimal/maternal non-optimal bonding combinations (OR: 1.72, 95 % CI: 1.10-2.68, *P* = 0.018) after adjustment for demographic variables and depression. The combination paternal optimal/maternal non-optimal was not significantly different (OR: 1.25, 95 % CI: 0.61-2.58, *P* = 0.538). The interaction effects between the parental bonding styles and the sex of the participants on the presence of chronic pain were not significant between paternal (*P* = 0.60) and maternal bonding (*P* = 0.68).

## Discussion

This study is the first report, as far as we know, that presents the relationship between parental bonding styles in childhood and the prevalence of chronic pain in adulthood in a general population. Lower levels of paternal care and higher levels of paternal and maternal overprotection were associated with an increased risk of chronic pain later in life in this community-based sample. Moreover, the PBI subtype analysis demonstrated that the group characterized by low care and high overprotection (“affectionless control”) was significantly more likely to have chronic pain than the group characterized by high care and low overprotection (“optimal bonding”) for both parents. Interestingly, paternal bonding was more robustly associated with the presence of chronic pain than was maternal bonding. After adjusting for depression, paternal bonding remained associated with the presence of chronic pain, but maternal bonding was not. These results suggest that the parental bonding style during childhood is related to the prevalence of chronic pain in adults in the general population. These associations were not significantly different between men and women.

A number of studies using the PBI have found that low care and high overprotection are linked with maladaptive characteristics in adults, such as low self-esteem [27], poorer interpersonal relationships [28–30], and difficulties coping with stress [31]. Reports also indicate that this PBI pattern contributes to several psychiatric disorders [32], including anxiety disorder [33], substance abuse, borderline personality disorder [34] and eating disorder [35]. The accumulated evidence demonstrates a particular association between adverse parental bonding and the development of depression in clinical and community samples [36, 37]. Depression is also recognized as a risk factor for chronic pain [38]. Supporting this, the current study found that the magnitude of the association between parental bonding and chronic pain attenuated after adjusting for depression. These findings collectively suggest that depression influences the link between parental bonding and chronic pain.

In the current study, the association between paternal bonding and chronic pain remained significant after the adjustment for depression, whereas the association with maternal bonding did not. These findings raise the possibility that maternal bonding may influence the prevalence of chronic pain mediated mainly by depression, while paternal bonding may have additional mechanisms. The reason for this difference between parents is currently unknown; however, it may be related to gender differences in parental influence on the development of a child’s coping strategies: Fathers are more likely to promote active, autonomous, and curious attitudes in children through encouraging their children to be independent, adventurous, and risk taking; whereas mothers place importance on emotional security and personal safety [39, 40]. The fact that fathers have been shown to be much more likely to use physical punishment and abuse than mothers in several epidemiological studies may also be related to these findings [41, 42]. Alternatively, the limited statistical power of this study may potentially explain the stronger effect of paternal bonding compared to that of maternal bonding; the median score for maternal care (31) was near the upper end of its range (36), which may have limited the ability to evaluate the effects of maternal care. Clearly, further studies are needed to elucidate the mechanism(s) whereby parental care affects the development of chronic pain in a gender-dependent fashion [34, 39].

This study has several limitations. First, the design is cross-sectional. As a result, no conclusions can be drawn regarding the causality or temporal order of the relationships. Namely, we cannot determine if parenting during childhood actually contributes to the development of chronic pain in adulthood or if the presence of chronic pain influences memories about the parenting actually received. Prospective longitudinal studies are needed to clarify the contribution of parenting to the development of chronic pain, although it will take several decades. Second, the assessment of parental bonding was based on a self-reported, retrospective measure. Although the PBI has been shown to be independent of the current mood state or life events [16] and has adequate test-retest reliability for a retrospective period of 20 years [18], we cannot rule out the possibility that recall bias and the current mood state during the study influenced the results. However, a perceived experience other than the objective manner of parents, such as harassment problems, might be more important in individual social pain experience. Third, a substantial number of residents participated in the Hisayama survey, but a little fewer than half agreed to participate in this survey. Therefore, selection bias may have influenced the results, and replication of the current findings using other community-based samples is needed. Fourth, most of the previous studies using PBI quadrants targeted relatively small clinical samples with mental problems. The available data on the distribution ratio of PBI quadrants in general population is limited. In most of these studies, assignment to high or low categories was conventionally based on the following cut-points: low care 24 for fathers and 27.0 for mothers and high overprotection 12.5 for fathers and 13.5 for mothers. These cut-points were determined on the basis of the mean scores of non-clinical Australian samples matched with depressive patients [36]. However, given the differences in culture and study population, it is not necessarily appropriate to apply the above-mentioned cut-points to the Japanese general population. Therefore, we used different cut-points based on this population. Further studies are needed in regards to the appropriate cut-points in consideration of chronic pain in the general population. Finally, we used only depression as a potential mediator between parenting and the presence of chronic pain. Therefore, measures of other possible relevant factors, such as anxiety and pain-related fear, coping, abuse experience, and other family-of-origin variables should be included in future studies.

## Conclusions

The results show, in a general population, that perceived parental care and overprotection during childhood are related to the risk of chronic pain in adulthood. Moreover, mass-education on parenting behaviors for optimal bonding and secure attachment may be one of the most promising preventive initiatives from the view of global health, beyond the concept of attachment. Clearly, further studies are necessary because the establishment of optimal bonding between child and parent through either the education of parents or the implementation of skill training programs may reduce the risk of the future development of chronic pain.

## References

[1] Andersson HI, Ejlertsson G, Leden I, Rosenberg C (1993). Chronic pain in a geographically defined general population: studies of differences in age, gender, social class, and pain localization. Clin J Pain.

[2] Tunks ER, Crook J, Weir R (2008). Epidemiology of chronic pain with psychological comorbidity: prevalence, risk, course, and prognosis. Can J Psychiatry.

[3] Turk DC, Okifuji A (2002). Psychological factors in chronic pain: evolution and revolution. J Consult Clin Psychol.

[4] Linton SJ (2002). A prospective study of the effects of sexual or physical abuse on back pain. Pain.

[5] Goodwin RD, Hoven CW, Murison R, Hotopf M (2003). Association between childhood physical abuse and gastrointestinal disorders and migraine in adulthood. Am J Public Health.

[6] Sachs-Ericsson N, Cromer K, Hernandez A, Kendall-Tackett K (2009). A review of childhood abuse, health, and pain-related problems: the role of psychiatric disorders and current life stress. J Trauma Dissociation.

[7] Jones GT, Power C, Macfarlane GJ (2009). Adverse events in childhood and chronic widespread pain in adult life: Results from the 1958 British Birth Cohort Study. Pain.

[8] Bowlby J (1977). The making and breaking of affectional bonds. I. Aetiology and psychopathology in the light of attachment theory. An expanded version of the Fiftieth Maudsley Lecture, delivered before the Royal College of Psychiatrists, 19 November 1976. Br J Psychiatry.

[9] Meredith PJ, Strong J, Feeney JA (2006). The relationship of adult attachment to emotion, catastrophizing, control, threshold and tolerance, in experimentally-induced pain. Pain.

[10] McWilliams LA, Asmundson GJ (2007). The relationship of adult attachment dimensions to pain-related fear, hypervigilance, and catastrophizing. Pain.

[11] McWilliams LA, Cox BJ, Enns MW (2000). Impact of adult attachment styles on pain and disability associated with arthritis in a nationally representative sample. Clin J Pain.

[12] MacDonald G, Kingsbury R (2006). Does physical pain augment anxious attachment?. J Soc Pers Relatsh.

[13] Meredith P, Strong J, Feeney JA (2006). Adult attachment, anxiety, and pain self-efficacy as predictors of pain intensity and disability. Pain.

[14] Parker G, Tupling H, Brown LB (1979). A parental bonding instrument. Br J Med Psychol.

[15] Ohmura T, Ueda K, Kiyohara Y, Kato I, Iwamoto H, Nakayama K (1993). Prevalence of type 2 (non-insulin-dependent) diabetes mellitus and impaired glucose tolerance in the Japanese general population: the Hisayama Study. Diabetologia.

[16] Parker G (1981). Parental reports of depressives. An investigation of several explanations. J Affect Disord.

[17] Parker G (1986). Validating an experiential measure of parental style: the use of a twin sample. Acta Psychiatr Scand.

[18] Wilhelm K, Niven H, Parker G, Hadzi-Pavlovic D (2005). The stability of the Parental Bonding Instrument over a 20-year period. Psychol Med.

[19] Parker G (1989). The Parental Bonding Instrument: psychometric properties reviewed. Psychiatr Dev.

[20] Kitamura T, Suzuki T (1993). A validation study of the Parental Bonding Instrument in a Japanese population. Jpn J Psychiatry Neurol.

[21] Kroenke K, Spitzer RL, Williams JB (2001). The PHQ-9: validity of a brief depression severity measure. J Gen Intern Med.

[22] Phelan E, Williams B, Meeker K, Bonn K, Frederick J, Logerfo J (2010). A study of the diagnostic accuracy of the PHQ-9 in primary care elderly. BMC Fam Pract.

[23] Krause JS, Reed KS, McArdle JJ (2010). Factor structure and predictive validity of somatic and nonsomatic symptoms from the patient health questionnaire-9: a longitudinal study after spinal cord injury. Arch Phys Med Rehabil.

[24] Muramatsu K, Miyaoka H, Kamijima K, Muramatsu Y, Yoshida M, Otsubo T (2007). The patient health questionnaire, Japanese version: validity according to the mini-international neuropsychiatric interview-plus. Psychol Rep.

[25] Hawker GA, Mian S, Kendzerska T, French M (2011). Measures of adult pain: Visual Analog Scale for Pain (VAS Pain), Numeric Rating Scale for Pain (NRS Pain), McGill Pain Questionnaire (MPQ), Short-Form McGill Pain Questionnaire (SF-MPQ), Chronic Pain Grade Scale (CPGS), Short Form-36 Bodily Pain Scale (SF-36 BPS), and measure of Intermittent and Constant Osteoarthritis Pain (ICOAP). Arthritis Care Res (Hoboken).

[26] Merskey H, Bogduk N (1994). Classification of Chronic Pain: Descriptions of Chronic Pain Syndromes and Definitions of Pain Terms.

[27] Parker G (1993). Parental rearing style: examining for links with personality vulnerability factors for depression. Soc Psychiatry Psychiatr Epidemiol.

[28] Otani K, Suzuki A, Shibuya N, Matsumoto Y, Kamata M (2009). Dysfunctional parenting styles increase interpersonal sensitivity in healthy subjects. J Nerv Ment Dis.

[29] Parker G, Barnett B (1988). Perceptions of parenting in childhood and social support in adulthood. Am J Psychiatry.

[30] Rodgers B (1996). Reported parental behaviour and adult affective symptoms. 2. Mediating factors. Psychol Med.

[31] Kraaij V, Garnefski N, De Wilde EJ, Dijkstra A, Gebhardt W, Maes S (2003). Negative life events and depressive symptoms in late adolescence: bonding and cognitive coping as vulnerability factors?. J Youth Adolesc.

[32] Enns MW, Cox BJ, Clara I (2002). Parental bonding and adult psychopathology: results from the US National Comorbidity Survey. Psychol Med.

[33] Silove D, Parker G, Hadzi-Pavlovic D, Manicavasagar V, Blaszczynski A (1991). Parental representations of patients with panic disorder and generalised anxiety disorder. Br J Psychiatry.

[34] Nickell AD, Waudby CJ, Trull TJ (2002). Attachment, parental bonding and borderline personality disorder features in young adults. J Pers Disord.

[35] Jáuregui Lobera I, Bolaños Ríos P, Garrido Casals O (2011). Parenting styles and eating disorders. J Psychiatr Ment Health Nurs.

[36] Parker G (1983). Parental ‘affectionless control’ as an antecedent to adult depression. A risk factor delineated. Arch Gen Psychiatry.

[37] Sato T, Sakado K, Uehara T, Narita T, Hirano S, Nishioka K (1998). Dysfunctional parenting as a risk factor to lifetime depression in a sample of employed Japanese adults: evidence for the ‘affectionless control’ hypothesis. Psychol Med.

[38] Mallen CD, Peat G, Thomas E, Dunn KM, Croft PR (2007). Prognostic factors for musculoskeletal pain in primary care: a systematic review. Br J Gen Pract.

[39] Sarkadi A, Kristiansson R, Oberklaid F, Bremberg S (2008). Fathers’ involvement and children’s developmental outcomes: a systematic review of longitudinal studies. Acta Paediatr Int J Paediatr.

[40] Paquette D (2004). Theorizing the father-child relationship: mechanisms and developmental outcomes. Hum Dev.

[41] Millichamp J, Martin J, Langley J (2006). On the receiving end: young adults describe their parents’ use of physical punishment and other disciplinary measures during childhood. N Z Med J.

[42] Sunday S, Labruna V, Kaplan S, Pelcovitz D, Newman J, Salzinger S (2008). Physical abuse during adolescence: gender differences in the adolescents’ perceptions of family functioning and parenting. Child Abuse Negl.

